# *Streptococcus pneumoniae* carriage studies in adults: Importance, challenges, and key issues to consider when using quantitative PCR-based approaches

**DOI:** 10.3389/fmicb.2023.1122276

**Published:** 2023-02-16

**Authors:** Willem R. Miellet, Sónia T. Almeida, Krzysztof Trzciński, Raquel Sá-Leão

**Affiliations:** ^1^Department of Pediatric Immunology and Infectious Diseases, Wilhelmina Children’s Hospital, University Medical Center Utrecht, Utrecht, Netherlands; ^2^Centre for Infectious Disease Control, National Institute for Public Health and the Environment (RIVM), Bilthoven, Netherlands; ^3^Laboratory of Molecular Microbiology of Human Pathogens, Instituto de Tecnologia Química e Biológica António Xavier, Universidade Nova de Lisboa, Oeiras, Portugal

**Keywords:** *Streptococcus pneumoniae*, colonization, adult, qPCR, nasopharynx, oropharynx, saliva, serotype

## Abstract

*Streptococcus pneumoniae* causes significant morbidity and mortality among older adults. Detection of pneumococcal carriage is an accepted endpoint in pneumococcal conjugate vaccine studies. However, low sensitivity of culture-based approaches and nasopharyngeal samples have hampered adult *S. pneumoniae* carriage studies in the past. In contrast, detection of adult *S. pneumoniae* carriers with qPCR-based approaches can achieve high sensitivity and specificity and qPCR-based testing of oral samples improves accuracy of adult carriage detection. In this Viewpoint we outline a strategy for accurate qPCR-based testing. We recommend a dual-target approach for *S. pneumoniae* qPCR detection as no genetic target is universally present among or solely unique to it. Furthermore, we advise the evaluation of concordance among quantified qPCR targets to improve the accuracy of *S. pneumoniae* testing and qPCR-based serotyping. We do not recommend omission of qPCR-based oral sample testing as it will likely result in an underestimation of true adult carrier rates.

## Introduction

*Streptococcus pneumoniae* is a leading cause of infectious diseases across the globe and a major disease burden of older adults (Bogaert et al., 2004; Collaborators, 2017). While 165 countries have implemented pneumococcal conjugate vaccines in infant immunization programs, fewer have classified old age as high-risk condition for pneumococcal disease. Until now, 54 have issued recommendations for adult immunization (Grant et al., 2021).

Pneumococcal conjugate vaccines (PCVs) target a subset of the known 101 capsular types and are protective against disease as well as pneumococcal colonization (Bentley et al., 2006; Ganaie et al., 2021b). As such, carriage is an accepted endpoint in vaccination studies (Auranen et al., 2013; Nzenze et al., 2017).

The World Health Organization (WHO) Pneumococcal Carriage Working Group published updated recommendations for standard methods for pneumococcal carriage studies in 2013 (Satzke et al., 2013). These guidelines have been used frequently worldwide (Turner et al., 2022). Carriage studies are particularly useful in populations where *S. pneumoniae* carriage is prevalent and occurs at high pneumococcal density, as is the case for nasopharyngeal colonization among young children (Bosch et al., 2016; Nunes et al., 2016; Félix et al., 2021). Very briefly, following the guidelines, nasopharyngeal swabs are obtained and inoculated onto selective agar medium. After incubation, presumptive pneumococcal colonies can be easily isolated, cultivated, and further characterized (Satzke et al., 2013). For adult studies, the additional use of oropharyngeal swabs has been recommended by the WHO (Satzke et al., 2013). However, a strictly culture-based approach to detect pneumococci in carriage samples from adults has repeatedly demonstrated insufficient sensitivity, resulting in low prevalence estimates (Almeida et al., 2014; Bosch et al., 2016; Southern et al., 2018; Arguedas et al., 2020).

In the last decade, there has been growing consensus that molecular methods are needed to avoid underestimation of adult pneumococcal carriage rates (Carvalho Mda et al., 2007; Satzke et al., 2013; Trzcinski et al., 2013; Krone et al., 2015; Van Deursen et al., 2016; Wyllie et al., 2016; Almeida et al., 2020, 2021). In this Viewpoint, we propose a strategy for accurate detection of *S. pneumoniae* carriage in adults based on quantitative PCR (qPCR).

## One hundred forty years of pneumococcal carriage studies

Pneumococci were first independently isolated by Louis Pasteur and George Sternberg in 1881 from saliva of asymptomatic carriers (Pasteur, 1881; Sternberg, 1881). By the early 1900’s, not only the role of *S. pneumoniae* as the main cause of bacterial pneumonia was firmly established, but “it was clear that pneumococci could be obtained from the mouths of 45–60 per cent on normal persons” (Heffron, 1939). Importantly, with lower carriage prevalence rates observed in children, a preponderance of those early studies on pneumococcal carriage have been conducted in adults using exclusively oral samples (Heffron, 1939). Of note, these early investigators also recorded that adults were more frequently found to carry *S. pneumoniae* in the throat than in the nasal passages (Heffron, 1939).

Until the mid-20th century, pneumococcal carriage was studied by resorting to animal inoculation as the gold standard in colonization detection (Rosenau et al., 1926; Heffron, 1939; Mackenzie, 1941). These early studies investigated transmission of virulent serotypes during outbreaks of pneumococcal pneumonia in crowded settings or described pneumococcal epidemiology for the general population with the purpose of improving pneumococcal pneumonia diagnostics (Heffron, 1939). The interest in carriage studies declined with the advent of antibiotics, only to rise again with the emergence and global spread of multidrug-resistant clones (Sá-Leão et al., 2000, 2002). The development and increased use of selective culture media has understandably led to abandonment of laboratory animal inoculation to detect human pneumococcal carriage (Converse and Dillon, 1977).

Research on pneumococcal carriage accelerated with the introduction of PCVs in the early 2000’s (Bogaert et al., 2004). Since PCVs protect vaccinees not only against disease but also against carriage of strains targeted by the vaccine (vaccine serotypes, VTs), carriage of *S. pneumoniae* became an endpoint in vaccine effectiveness studies (Auranen et al., 2013; Nzenze et al., 2017). This included randomized controlled trials on PCVs’ direct effects in children (Dagan et al., 1997; van Gils et al., 2009) and surveillance of carriage to complement studies on PCVs’ impact on pneumococcal disease (IPD) (Nunes et al., 2016; Lewnard et al., 2020; Félix et al., 2021).

## Why study pneumococcal carriage in adults?

For the past two decades, pneumococcal carriage studies in adults have been relatively scarce due to the assumption that adult carrier rates are very low. With the increasing availability of PCVs for adult use, and their ongoing incorporation in adult immunization plans, studies on carriage in non-pediatric populations are gaining importance as they can be quite informative as highlighted in Table 1.

**Table 1:** Potential outcomes of pneumococcal carriage studies.

Outcomes	References^1^
Direct effects of vaccine	Kandasamy et al. (2019) and Félix et al. (2021)
Replacement of vaccine serotype (VT) strains by non-vaccine serotype (NVT) strains	Kandasamy et al. (2019) and Félix et al. (2021)
Secular trends and impact of PCVs on pneumococcal diversity	Paulo and Sá-Leão (2017, 2021)
Estimation of serotype-associated invasive disease potential	Balsells et al. (2018)
Surveillance of changes occurring in virulence and antimicrobial resistance	Almeida et al. (2014) and Tin Tin Htar et al. (2019)
Identification of risk factors associated with adult carriage	Krone et al. (2015), Wyllie et al. (2016), Almeida et al. (2020, 2021), and Miellet et al. (2021)
Tracking of asymptomatic transmission in the general population and during outbreaks of pneumococcal disease	Amin-Chowdhury et al. (2019)
Determination of colonization parameters (rates of strain acquisition and clearance, duration of carriage, R0, etc.)	Tigoi et al. (2012) and Almeida et al. (2021)
Interactions between pneumococci and other microorganisms (including viruses) sharing the same niche	Miellet et al. (2021) and Lewnard et al. (2022)
Evaluation of the potential of non-pharmaceutical interventions to prevent transmission	Danino et al. (2022)
Improvement of diagnostics of pneumococcal pneumonia	Carr et al. (2022)

^1^Cited studies, whenever possible, included adult populations.

## Strategies based on culture alone are often insufficient for sensitive pneumococcal carriage detection in adults

As oral samples are highly polymicrobial, and pneumococci are generally present at low absolute and relative abundances (Miellet et al., 2021, 2022), isolation of viable pneumococci from oropharyngeal and saliva samples is often very difficult, if not nearly impossible (Carvalho Mda et al., 2013; Wyllie et al., 2014; Krone et al., 2015; Simões et al., 2016; Wyllie et al., 2016). By contrast in the nasopharynx, particularly in children, pneumococci tend to be present at high density and isolation of viable pneumococci is straightforward (Levine et al., 2012; Arguedas et al., 2020; Almeida et al., 2021). Consequently, with the increased use of selective culture media, the nasopharynx became the preferential site to sample for pneumococci, and the focus in carriage studies shifted almost entirely toward children.

In fact, studies in adults based solely on cultivation of nasopharyngeal and or oropharyngeal samples on selective agar media have mostly documented very low pneumococcal carriage rates (<5%) (Regev-Yochay et al., 2004; Flamaing et al., 2010; Almeida et al., 2014; Miellet et al., 2022). This may have led to the erroneous notion that adult pneumococcal carriage is rare. Poor sensitivity of conventional culture when applied to oral fluids and oropharyngeal swabs is a limitation that, nowadays, can be overcome with molecular diagnostic methods. The latter are also useful for the detection of multiple serotype carriage events which is challenging with culture-based approaches (Huebner et al., 2000; Valente et al., 2013). This can be particularly useful, for example, to unmask potential carriage of minor serotypes, such as vaccine types in settings where PCVs have been widely used (Valente et al., 2016).

## A proposal for accurate sensitive detection of adult pneumococcal carriage based on selective culture and qPCR

Available molecular methods, mostly based on qPCR, can be used for the detection of pneumococci with high sensitivity and specificity (Carvalho Mda et al., 2007; Azzari et al., 2010; Trzcinski et al., 2013; Wyllie et al., 2017). Among those, detection of *lytA* and *piaB*, and more recently, SP2020, are increasingly used (Carvalho Mda et al., 2007; Trzcinski et al., 2013; Tavares et al., 2019).

With high accuracy and mirroring pneumococcal prevalence rates observed with historical methods, advances in molecular methods have made adult carriage studies again feasible. There is, however, the need to have a robust experimental strategy to maintain high specificity while achieving high sensitivity of pneumococcal detection in polymicrobial samples.

**Figure 1:**
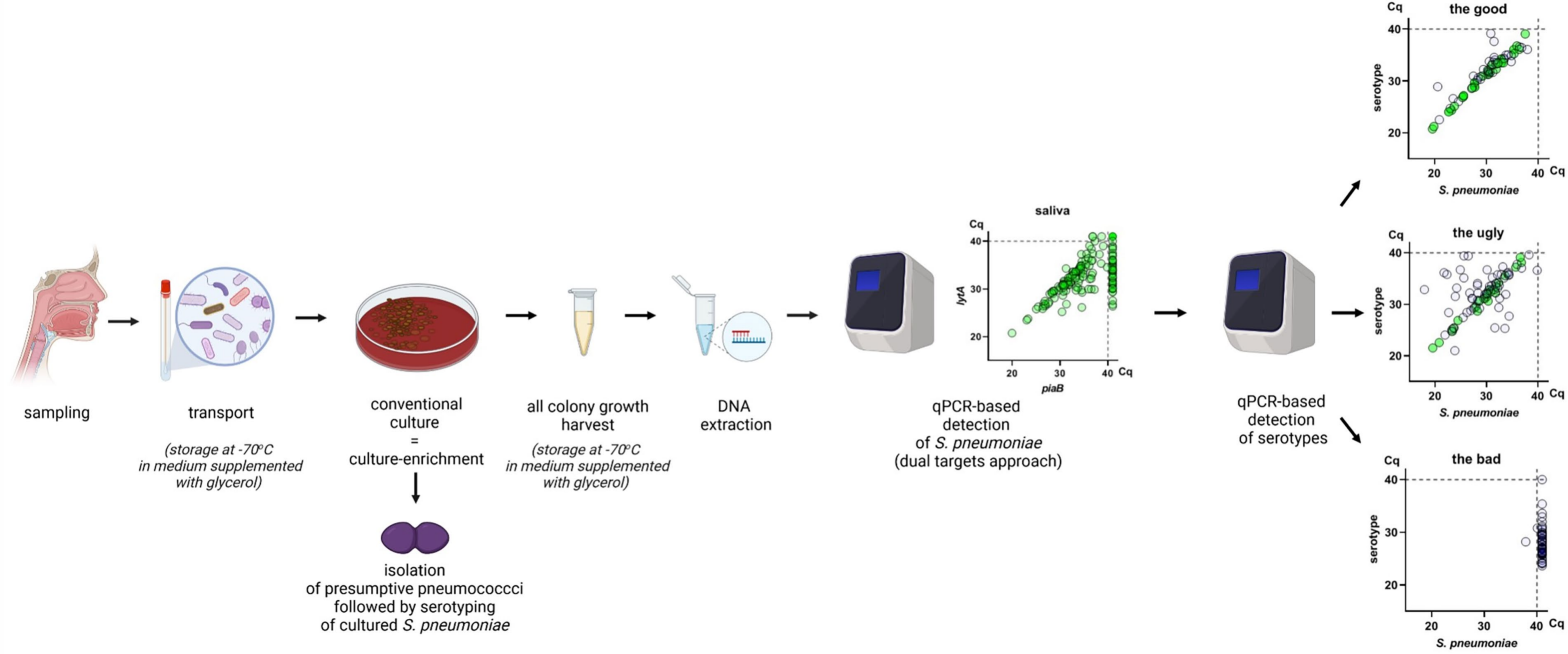
Visual summary of the “good” protocol. A nasopharyngeal, oropharyngeal or saliva sample is collected from a study participant and transported in medium supplemented with 10% glycerol. The sample is cultured on defibrinated sheep blood agar supplemented with gentamicin. Presumptive *S. pneumoniae* colonies are isolated for detection by conventional culture. Then, all microbial growth on the culture plate is harvested with a 10 μl inoculation loop or by washing the plate with a liguid medium and the cells suspension is stored in medium supplemented with 10% glycerol. DNA extraction is performed on the culture-enriched harvest and molecular detection is conducted using a dual-target approach with *lytA* and *piaB* or *lytA* and SP2020 (*bguR*). Concordance between quantified qPCR targets is evaluated and samples are classified as positive or negative by qPCR. Samples negative for *S. pneumoniae* by qPCR are pooled by ten and positive samples are pooled by five. Pools are tested with serotype/serogroup-specific qPCR assays. The specificity of serotype/serogroup-specific assays is evaluated using pools from negative samples. Pools from positive samples are tested for serotype/serogroup-specific qPCR assays, when a pool is positive for a particular assay all samples from that pool are individually tested. The figure was made in BioRender.

In the next sections we outline key factors of an experimental protocol for accurate identification of pneumococcal carriage using qPCR-based approaches (Figure 1). This should allow for routine detection of adult pneumococcal carriage and serotype assignment and is particularly suited for studies using oral samples. For a detailed experimental protocol see also the Supplementary Text.

As supplementary material, we advise on procedures, in particular statistical methods, complementing the qPCR-based protocol which allow for increased accuracy of carriage detection (Supplementary Figure S1). The latter strategy (i) improves the specificity of qPCR-based detection by reducing impact of relic DNA using data-driven *C*_q_ cut-offs, (ii) improves sensitivity of culture by using qPCR-guided culturing, (iii) helps to classify serogroup/serotype-specific qPCR assays as reliable or non-reliable using Bland–Altman plots (Supplementary File), (iv) improves comparison of methods (culture vs. qPCR), and (v) allows accurate comparison of sample types. Hence, this information may be of interest for researchers willing to accommodate extra scrutiny into their studies or using novel approaches for pneumococcal carriage detection.

## Accurate identification of pneumococcal carriage using qPCR-based approaches: Key issues

### Sampling, transport and storage

While for children there is a consensus that sampling of the nasopharynx is adequate for pneumococcal carriage detection, for adults this matter is debated. Recent comparative studies in adults show that rates of carriage detected with qPCR in oropharyngeal samples are higher than in nasopharyngeal swabs (Krone et al., 2015; Wyllie et al., 2016; Almeida et al., 2021; Miellet et al., 2022). The qPCR-guided culturing also rendered oropharyngeal samples to be more sensitive than nasopharyngeal samples (Supplementary Text; Wyllie et al., 2014, 2016; Miellet et al., 2022). Of interest, a study conducted among healthy adults in Portugal (Almeida et al., 2021) did not find saliva samples to be superior for *S. pneumoniae* detection unlike Dutch studies where saliva testing with qPCR outperformed nasopharyngeal and oropharyngeal swabs (Krone et al., 2015; Wyllie et al., 2016). Importantly, these studies indicate that sampling multiple sites may be beneficial and that the use of qPCR-based methods is of advantage to study pneumococcal carriage as it increases the sensitivity of carriage detection (Trzcinski et al., 2013; Wyllie et al., 2016; Almeida et al., 2020, 2021; Miellet et al., 2022).

When choosing a sample type to be collected and tested for pneumococcal carriage, both the accuracy and practical use of the approach need to be considered. While nasopharyngeal and oropharyngeal swabs need to be collected by a trained health professional (Satzke et al., 2013), saliva can be self-collected by either spitting or using a sponge-made device (Supplementary Text; Wyllie et al., 2014; Krone et al., 2015; Almeida et al., 2021). Unlike nasopharyngeal and oropharyngeal swabs, saliva does not require a transport medium (e.g., STGG or Amies) (van Gils et al., 2009), but it does need a ‘cold chain’: transport on dry-ice (after supplementing with glycerol) or, if cultured upon arrival in the lab, transport on wet ice (Krone et al., 2015; Almeida et al., 2021). To preserve viable pneumococci during storage at ≤70°C, a sample needs to contain 10–20% glycerol.

Alternatively, studies focusing exclusively on molecular detection of pneumococci (with no intention of culturing viable cells), can resort to widely used transport media that preserve nucleic acids (such as those containing guanidine isothiocyanate).

### Culture-enrichment and DNA extraction

Culture-enrichment should be conducted to enhance the sensitivity of molecular detection and reduce the presence of other microorganisms (and hence non-pneumococcal DNA) within a sample. In addition, culture-enrichment also contributes to reducing relic DNA in samples (Wyllie et al., 2014; Lennon et al., 2018; Miellet et al., 2021, 2022). A commonly used medium for pneumococcal culture-enrichment is defibrinated blood agar supplemented with gentamicin (Satzke et al., 2013). As absolute and relative pneumococcal abundances are often greatly reduced in oral when compared to nasopharyngeal samples (Miellet et al., 2022), for oral samples, processing a larger volume is advised to maintain sufficient sensitivity of detection.

DNA extraction of pneumococcal culture-enriched samples should be carried out in conditions that prevent in-house contamination. Culture-enriched frozen stocks should be opened in a laminar flow cabinet under sterile conditions to prepare aliquots (typically of 200 μl) for DNA extraction. DNA extraction should be carried out in a separate clean room preferably using a closed automated system. To avoid potential batch effects, when processing samples, one should avoid separating samples by study groups. However, separation of minimally processed samples from culture-enriched samples, and separation of culture-enriched samples by specimen type (e.g., process culture-enriched nasopharyngeal samples separately from culture-enriched saliva), may be appropriate. Immediately prior to extraction of DNA from culture-enriched saliva samples, potential PCR inhibitory compounds should be inactivated *via* a heating step (e.g., 15 min at 95°C). For each batch of DNA extraction, as a negative control, ultrapure water should be processed in parallel with the samples under investigation.

### qPCR-based detection in culture-enriched sample

**Figure 2:**
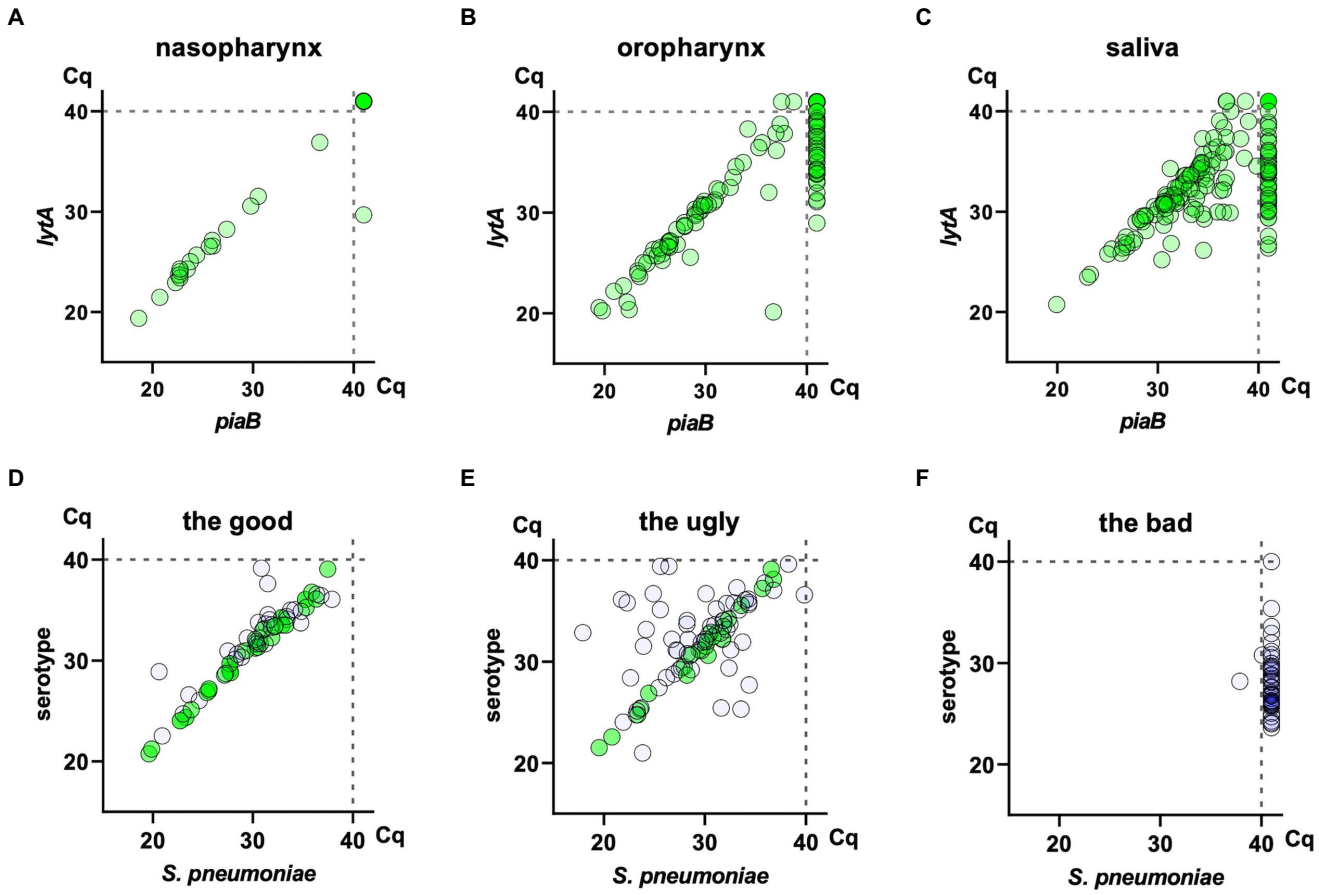
Detection of *Streptococcus pneumoniae* and pneumococcal serotypes with molecular methods in nasopharyngeal, oropharyngeal and saliva samples collected in 2014/2015 from asymptomatic individuals in The Netherlands (Miellet et al., 2022; Submitted).^1^ The top three panels depict scatter plots of *piaB* and *lytA* qPCR cycle threshold (*C*_q_) values for nasopharyngeal **(A)**, oropharyngeal **(B)**, and saliva **(C)** samples collected from *n* = 322 adults. In all six panels, each symbol represents an individual sample. The three bottom panels depict scatter plots of pneumococcus-specific qPCR (here *piaB*, *X*-axis) and serotype-specific qPCR (*Y*-axis) cycle threshold (*C*_q_) values for assays targeting serotypes 23B **(D)**, 19A **(E)**, and 5 **(F)** applied to *n* = 975 saliva samples collected in the same study from *n* = 322 adults and *n* = 653 children aged 2–4  years. Samples with a *C*_q_ > 40 in serotype-specific qPCR **(D–F)**, hence negative for a serotype are not shown. Here, green dots represent saliva samples from individuals from whom a strain of the corresponding serotype was cultured: in case of children either nasopharyngeal or saliva sample, in case of adults either nasopharyngeal, oropharyngeal or saliva sample. Remaining dots represent samples from individuals for whom no viable *S. pneumoniae* of the corresponding serotype could be recovered *via* culture. **(D)** Depicts results for serotype-specific assays considered highly reliable based on the absence of samples with a signal for serotype stronger than that for *S. pneumoniae*, hence classified as “the good.” **(E)** Depicts results for serotype-specific assays of reduced specificity based on the occurrence of a serotype signal stronger than the one obtained for the *S. pneumoniae* specific gene, hence classified as “the ugly.” Here, despite the reduced specificity of the serotype-specific assay, samples of *C*_q_ for a serotype closely matching the *C*_q_ for *S. pneumoniae* represent reliable positive results. **(F)** Depicts results for serotype-specific assays deemed unreliable based on the positivity of samples classified as negative for *S. pneumoniae*, hence classified as “the bad.” Consequently, the samples positive for *piaB* and *lytA* were not tested with qPCR for that serotype.

For qPCR detection of *S. pneumoniae*, at least two genetic determinants should be targeted using validated primers and probes. We recommend the combined use of *lytA* (Carvalho Mda et al., 2007) and *piaB* (Trzcinski et al., 2013) or, alternatively, *lytA* and SP2020 (*bguR*) (Tavares et al., 2019). Of note, individually, none of these targets are ideal, i.e., none is universally present in *S. pneumoniae* and universally absent in other bacterial species (Wyllie et al., 2017; Tavares et al., 2019). Still, when used in combination (Figures 2A–C), very high sensitivity and specificity for pneumococcal detection are obtained (Tavares et al., 2019; Miellet et al., 2022). Two-step and multi-step bacteriological identification procedures have long been part of the standard practice for clinical microbiological laboratories (Mundy et al., 1998); qPCR-based techniques are no exception, in particular for promiscuous bacteria such as *S. pneumoniae*.

A key factor when using this dual-target approach is to inspect qPCR results for agreement (correlation without bias) between *C_q_* values obtained for each of the targets (for further information see Supplementary Text). As *lytA*, *piaB*, and SP2020 rarely occur in multiple copies in the pneumococcal genome, and the qPCR assays targeting these genes are highly efficient, for each pneumococcal culture, concordant *C*_q_s are expected. In other words, if a sample is found to provide concordant *C*_q_s for both targets, there is high confidence in that it contains pneumococci (Trzcinski et al., 2013; Wyllie et al., 2016; Almeida et al., 2020, 2021; Miellet et al., 2022). As a rule of thumb, we define concordance between *C*_q_s from different targets as a difference of 2 *C*_q_s or less (for example, |*C*_q_ *lytA* – *C*_q_ *piaB*| ≤2). As pneumococci occasionally lack *piaB* (mostly non-encapsulated strains, but also some specific encapsulated lineages), *C*_q_ of *lytA* can be >2*C*_q_ lower (hence the signal stronger) compared to *C*_q_ of *piaB*. If relevant, one should consider testing such samples for SP2020. Notably, we have never identified a pneumococcus simultaneously missing two of these three targets. We advocate publishing figures displaying the degree of concordance in pneumococcal quantification (either *C*_q_s, DNA concentrations or number of genome copies) with complementary qPCRs (e.g., *lytA* vs. *piaB*, *lytA* or *piaB* vs. serotype).

To reduce interlaboratory variation and improve the specificity of detection by reducing detection of relic DNA, a study-specific and non-arbitrary *C*_q_ cut-off can be considered (see Supplementary Text).

As a positive control for samples undergoing culture enrichment, DNA extraction and qPCR-based detection of pneumococci, an aliquot of respiratory sample spiked with *S. pneumoniae* can be used. For this, respiratory samples from multiple volunteers can be pooled to generate enough aliquots to be used throughout the whole study.

Positive and negative controls should be included for all steps as described before (Carvalho Mda et al., 2007; CDC, 2016; Almeida et al., 2021). In each batch of qPCR reactions, positive (for example, *S. pneumoniae* TIGR4), negative (for example, *S. pseudopneumoniae* strain ATCC BAA-960), and non-template (ultrapure water) controls should be included. For further information regarding optimization of qPCR assays, see Supplementary Text.

### qPCR-based serotyping

A major endpoint of vaccine studies is assessing vaccine-serotype prevalence rates in carriage. Serotyping by qPCR is a culture-independent method wherein samples positive for *S. pneumoniae* by *piaB* and *lytA*specific qPCRs are subjected to serotype-specific qPCRs.

In case of individual serotype carriage, serotype-specific *C*_q_s should be concordant with *piaB* and *lytA C*_q_s (differing in 2 *C*_q_s or less) (Figures 2E,F). Samples containing multiple serotypes will exhibit lower serotype-specific signal (higher *C*_q_) when compared with *piaB* and *lytA*.

Careful interpretation of any test result is important as a negative nasopharyngeal sample does not necessarily preclude a positive oropharyngeal sample, and vice versa. Hence, lack of positive results in serotype-specific qPCRs (of validated schemes) should be interpreted as absence of that serotype exclusively in that tested sample.

We suggest that in all studies, samples negative for *S. pneumoniae* should be tested in parallel with positive samples, to evaluate the specificity of serotype-specific qPCRs. A serotype-specific qPCR assay should be considered non-reliable when a considerable number of *S. pneumoniae* negative samples are positive for a serotype-specific assay. Poor specificity in qPCR assays has been found for serotypes 4, 5, 9A/N/V, 12A/B/F, 22A/F, 23A, and 35B (Carvalho Mda et al., 2013; Krone et al., 2015; Wyllie et al., 2016; Almeida et al., 2020, 2021). Insufficient specificity can occur when non-pneumococcal streptococci carry homologues of pneumococcal capsular genes and thus are source of false positives (Carvalho Mda et al., 2012, 2013; Skov Sørensen et al., 2016; Wyllie et al., 2017; Lessa et al., 2018; Pimenta et al., 2018; Nahm et al., 2020; Ganaie et al., 2021a; Gertz et al., 2021). Specificity of serotype-specific qPCRs can be evaluated using Bland–Altman analysis (Supplementary Text). To overcome specificity problems whole genome sequencing analysis of pneumococcal and non-pneumococcal strains can be used to design alternative primers and/or probes (Velusamy et al., 2020).

To avoid testing a substantial number of samples for multiple serotype-specific qPCRs, samples can be pooled. For example, *S. pneumoniae* positive samples can be pooled by five and negative samples pooled by ten (Wyllie et al., 2016; Almeida et al., 2021; Miellet et al., 2022). Using this approach, once a positive signal is obtained for a given serotype, individual samples are re-tested for that serotype. Alternatively, serotype-specific assays can be multiplexed (Pimenta et al., 2013).

When concordant *C*_q_s for *lytA*, *piaB*, and a specific serotype are obtained for a given sample, there is a high confidence in that it contains pneumococci (Trzcinski et al., 2013; Wyllie et al., 2016; Almeida et al., 2020, 2021).

Important limitations of qPCR-based serotyping are lack of serotype-discrimination for several serogroups and presence of various capsular genes among non-pneumococcal Streptococci, and in oral samples in particular. The latter may hamper interpretation of results (Carvalho Mda et al., 2012, 2013; Pimenta et al., 2018) and even render certain serotype-specific qPCR assays unreliable (Supplementary Figure S2). Importantly, this limitation is not unique to qPCR-based serotyping as it has also been observed with urinary antigen tests and when using serotype-specific antisera (Austrian, 1973; Stralin et al., 2004). For the latter, however, as a cultured sample is needed, additional tests can be done for species assignment.

Notably, in Portuguese studies looking at multiple sample sites, whenever a pneumococcal culture was obtained from one site (for example, nasopharynx), and positive qPCR-based results were obtained from another site (for example, oropharynx or saliva), there was concordance in the serotypes determined (Almeida et al., 2020, 2021).

## Conclusion

Since there is a growing interest in *Streptococcus pneumoniae* carriage studies in adults we identified a number key points to be considered (Table 2). To summarize, poor sensitivity of culture-based approaches and nasopharyngeal swabs have most likely underestimated true adult carriages rates in the past while the presence of pneumococcal genes among non-pneumococcal streptococcal species in oral samples have discouraged some from using qPCR-based approaches. When faced with these two key issues that hinder informative adult pneumococcal carriage studies, we regard the latter issue to be the lesser of two evils and one that is surmountable with a robust experimental strategy. Molecular approaches, particularly those based on qPCR, are highly suited for adult pneumococcal carriage detection and can produce accurate and sensitive results. We hope that this Viewpoint contributes to clarify key aspects of sample preparation, testing and results interpretation.

**Table 2:** Key points for *Streptococcus pneumoniae* adult carriage studies.

Key points
Pneumococcal carriage is an accepted endpoint in vaccination studies.
Culture-based approaches and nasopharyngeal samples often display insufficient sensitivity for detection of adult *S. pneumoniae* carriers.
Testing of multiple sampling sites improves accuracy of carriage detection.
Adult *S. pneumoniae* carriers can be detected with high sensitivity and specificity with qPCR-based approaches.
Testing of oral samples with qPCR-based methods improves sensitivity of adult *S. pneumoniae* carriers.
No genetic target is universally present in or unique for *S. pneumoniae*.
A dual-target approach should be applied to qPCR-based detection of *S. pneumoniae* to maintain high accuracy of detection.
The evaluation of concordance in qPCR quantification can improve accuracy of pneumococcal detection and qPCR-based serotyping.
Omission of qPCR-based oral sample testing results in an underestimation of true adult carriage rates.

## Data availability statement

The original contributions presented in the study are included in the article/Supplementary material, further inquiries can be directed to the corresponding authors.

## Author contributions

KT and RS-L had the idea of writing this Viewpoint based on a Meet the Experts Session organized by both at the International Symposium of Pneumoccocci and Pneumococcal Diseases – ISPPD2022. All authors discussed the contents of the manuscript, actively contributed to its draft versions and final versions.

## Funding

The authors declare that this study received funding from GlaxoSmithKline Biologicals SA. The funder was not involved in the study design, collection, analysis, interpretation of data, the writing of this article, or the decision to submit it for publication. Funding for this study was provided in part (SA and RS-L) by LISBOA-01-0145-FEDER (Microbiologia Molecular, Estrutural e Celular, funded by FEDER through COMPETE2020 – Programa Operacional Competitividade e Internacionalização), the authors received no other form of compensation or financial support related to the development of the manuscript.

## Conflict of interest

KT reports receiving consultation and speaking fees and funds for unrestricted research grants from Pfizer, and funds for unrestricted research grants and consultation fees from Merck Sharp & Dohme, all paid directly to his home institution and none related to the present work. RS-L reports receiving consultation and speaking fees from Pfizer and consulting fees from Merck (US). RS-L reports also receiving funds for unrestricted research grants from Pfizer and Merck Sharp & Dohme not related to the present work, paid directly to her home institution.

The remaining authors declare that the research was conducted in the absence of any commercial or financial relationships that could be construed as a potential conflict of interest.

## Publisher’s note

All claims expressed in this article are solely those of the authors and do not necessarily represent those of their affiliated organizations, or those of the publisher, the editors and the reviewers. Any product that may be evaluated in this article, or claim that may be made by its manufacturer, is not guaranteed or endorsed by the publisher.

## Footnotes

^1^Miellet, W. R., and Trzciński, K. (2022). A Spitting Image: Molecular Diagnostics Applied to Saliva Enhance Detection of Streptococcus pneumoniae and Pneumococcal Serogroup/Serotype Carriage. [Submitted].
