# Supplementary material for: Streptococcus pneumoniae carriage studies in adults: Importance, challenges, and key issues to consider when using quantitative PCR-based approaches
Source: Front Microbiol. 2023 Feb 16;14:1122276. doi: 10.3389/fmicb.2023.1122276 (PMC9994646; doi:10.3389/fmicb.2023.1122276)
Supplement: Supplementary file 1 [file Data_Sheet_1.docx]

**Supplemental Text**

**Saliva sample collection, transport, culture and storage**

Saliva can be collected using a sponge-made device (Krone et al., 2015)or by spitting into a tube (Wyllie et al., 2014, Almeida et al., 2021). In particular, for saliva, we suggest collecting 400 μl-1ml to which glycerol is immediately added for a final concentration of 10-20%. We suggest transporting saliva on wet ice. Upon arrival in the laboratory, 50-100 μl of the mixture should be plated on selective agar medium for culture enrichment. Presumptive *S. pneumoniae* colonies can be isolated for detection by conventional culture (Satzke et al., 2013). For saliva samples, however, it is rare that pneumococcal cultures can be isolated at this stage. All visible microbial growth on the culture plate should be harvested with an inoculation loop, stored in medium supplemented with 10% glycerol and frozen at -70^o^C until further use. Colony growth can be also harvested by washing the plate with 2 ml of Brain Heart Infusion (Oxoid) supplemented with 10% glycerol, scraping off bacterial growth with a disposable spreader and mixing the cell suspension by repeated pippeting (Trzcinski et al., 2013). This bacterial growth will be used in qPCR for detection of pneumococci.

**Advanced Protocol for Molecular Detection of *S. pneumoniae***

*Preparing and Conducting qPCR-based Detection of S. pneumoniae*

In general, we recommend following the MIQE Guidelines when conducting qPCR experiments (Bustin et al., 2009). Very briefly, optimization of qPCR assays can be performed by testing primer combination concentrations (including asymmetric concentration combinations) in a matrix layout, testing various master mixes, performing amplicon size analysis and testing a probe concentration range. When optimizing qPCR assays in your laboratory it is advised to calculate the PCR standard curve amplification efficiency (henceforth: PCR efficiency) and R^2^. The PCR efficiency (Efficiency = 10^(-1/Slope)-1^) is of particular importance as the C_q_ values are dependent on PCR efficiency (Bustin et al., 2009). A PCR efficiency between 90% and 110% is ideal (Rogers-Broadway and Karteris, 2015). When multiplexing qPCR assays it is also important to evaluate the PCR efficiency to account for differences in primer efficiencies between multiplexed assays (Bustin et al., 2009). Of note, the efficiency of PCR reactions may markedly differ between the standard curve and samples (e.g. due to PCR inhibitory compounds). To rule out such a bias, a standard curve-independent method to calculate the reaction efficiency can also be considered (Ramakers et al., 2003).

A consistent and user friendly method to calculate the C_q_ is to use the second derivative maximum (SDM) C_q_ method (Miellet et al., 2020, Miellet et al., 2022). This method can reduce variation between qPCR assay experiments and is suited for high-throughput testing (Tellinghuisen and Spiess, 2019). However, substantial background fluorescence levels may lead to inaccuracies in C_q_ calculation (Ruiz-Villalba et al., 2021). Alternative C_q_ methods such as quantification thresholds based on a fixed amount of fluorescence can also be considered, as discussed elsewhere (Tellinghuisen and Spiess, 2019). However the usage of quantification thresholds can result in inaccurate C_q_s when plateau levels differ between samples (e.g. due to a probe mismatch) (Ruiz-Villalba et al., 2021). This may occur with various serotype or serogroup-specific qPCR assays. Most issues can be addressed during assay optimization and otherwise identified by routinely evaluating C_q_ variation in the standard curves from different qPCR experiments.

Brief curation of qPCR data, in particular of the standard curve and NTC prior to sample result interpretation is advised. The R^2^ from technical replicates of the standard curve can be used to check for PCR inhibitors and pipetting errors. An R^2^ <0.985 can be indicative of such issues (Rogers-Broadway and Karteris, 2015). When using a dual-target approach, both qPCR targets (e.g., *piaB* and *lytA*) can be considered as independent replicates of each other. This strategy can reduce costs of sample testing.

*Interpretation of C_q_ Results*

The certainty of a qPCR result increases as the C_q_ decreases and highly positive samples (e.g., C_q_ of 20) provide strong evidence of viable pneumococci presence. The presence of relic DNA (Lennon et al., 2018), in particular among raw (non-culture-enriched) samples can generate high C_q_s in otherwise negative samples. Here, relic DNA is defined as extracellular DNA present in the sample which origin is not from viable bacterial cells. It can be due to residual DNA present in the sample of a participant who has been exposed to the qPCR-targeted bacteria (but is not a carrier at the time of sampling), or due to background laboratory contamination. To limit this issue when encountered, a C_q_ cut-off such as < 35 C_q_ can be implemented (Wyllie et al., 2016, Boelsen et al., 2020). When processing samples for both culture-based detection and qPCR-based detection we propose to conduct a receiver operating characteristic (ROC) curve analysis to estimate C_q_ cut-off values per assay with isolation of live pneumococcus as criterium (**Figure S2**) (Miellet et al., 2021, Miellet et al., 2022). ROC curve analysis can improve the specificity of qPCR-based methods by implementing a non-arbitrary threshold, in this case the C_q_ value, with which a sample can be classified with high diagnostic accuracy. ROC curve analysis requires a criterium, such as culture, to be used for binary classification. In addition, C_q_ values from culture-positive and culture-negative samples are used in the analysis. ROC curve analysis then determines the C_q_ cut-off corresponding to the maximum value of Youden’s index (J = sensitivity + specificity - 1), representing the threshold with an optimal combination of sensitivity and specificity.

*Visualizing and Reporting qPCR Results*

When performing dual-target detection of *S. pneumoniae*, qPCR results can be plotted in scatter plots (**Figure 2**). As scatter plots can aid interpretation of data, also when qPCR detection is used for non-quantitative purposes, we recommend including scatter plots when reporting qPCR data. The position of the plotted data within such a figure can inform the researcher about differences in the quantification of qPCR targets (Ranganathan et al., 2017), such as bias between assays or outliers. The scatter plot can also be used to optimize qPCR assays and to evaluate sensitivity and specificity of new targets. A scatter plot can be complemented with an intraclass correlation coefficient (ICC; two-way mixed-effects model, single rater/measurements, consistency (Koo and Li, 2016, McGraw and Wong, 1996), (Shrout and Fleiss, 1979)) to assess quantitative agreement (reliability) between qPCR targets among positive samples (Miellet et al., 2022). Unlike Pearon’s r and Spearman’s ρ, the ICC assumes a common population variance among the two qPCR targets as they are presumed to measure the same target organism. Accordingly, this method can be used as an indicator of agreement by detecting differences in qPCR target variance while taking a systematic error into account (e.g. small differences in PCR assay efficiencies). This type of agreement, termed ‘consistency’ can be written as *piaB* C_q_ = *lytA* C_q_ + systematic error (McGraw and Wong, 1996). When sample size is deemed sufficient, the 95% CI of the ICC can be used to interpret reliability, by classifying <0.50, 0.50 to 0.75, 0.75 to 0.90, and >0.90 as indicative of poor, moderate, good and excellent reliability, respectively (Koo and Li, 2016). The ability of qPCR targets to classify sample status (e.g., positive or negative for pneumococcal presence) can also be compared using Cohen’s kappa (McHugh, 2012, Miellet et al., 2022).

Bland-Altman analysis can be performed for a more detailed visualization of agreement between qPCR target measurement among samples positive for both targets (**Figure S3**). In a Bland-Altman plot the mean difference between C_q_ of qPCR targets and the corresponding lower and upper limits of agreement (standard deviations of the mean difference) are plotted and can indicate bias. Significant bias between assays is observed when the 95% CI of the mean difference between qPCR targets does not overlap with the line of equality (0 bias) (Bland and Altman, 1986, Giavarina, 2015, Miellet et al., 2022). Bland-Altman analysis can also be used to easily identify outlier measurements from samples, as outliers are often plotted outside the limits of agreement (and corresponding 95% CIs).

Bland-Altman plots can be readily made in analytical software such as Graphpad Prism, R (using the ‘blandr’ package), IBM SPSS Statistics, and Excel (**supplemental Excel file**). A Bland-Altman plot can be complemented with a regression line to evaluate if bias is dependent on the C_q_ value.

*Bland-Altman Analysis with Serotype-specific qPCR Assays*

Serotype-specific qPCR assays can be evaluated in Bland-Altman analysis and used to classify assay as reliable or nonreliable. To this end, the limit of agreement between two targets (e.g. *piaB* and *lytA*) is calculated and can be used as an *a priori* acceptable limit to classify serotype-specific assays and individual measurements (the upper limit of agreement is used when the serotype assay is used as a second rater in Bland-Altman analysis and *piaB/lytA* as first rater) as reliable or nonreliable. When serotypes-specific assays consistently yield lower C_q_ values than *piaB* or *lytA* and these C_q_ measurements are plotted beyond the upper limit of agreement and its corresponding 95% CI (**supplemental Excel file**). However, the added value of this approach is dependent on the sample size of positive samples, therefore we strongly recommend testing serotype-specific assays with a panel of negative samples as described in the main text.

*qPCR-guided culturing*

Culture-enriched samples classified as negative in primary diagnostic culture yet positive by qPCR can be revisited with culture in a second attempt to isolate live pneumococci. For this, Columbia blood agar (CBA) plates are usually inoculated with 100 μl of 10^−3^-10^−5^ dilutions of culture-enriched saliva samples (10^−1^-10^−3^ dilutions in case of culture-enriched nasopharyngeal, and 10^−3^-10^−5^ dilutions in case of culture-enriched oropharyngeal samples). After plates overnight incubation at 37◦C and 5% CO_2_ the pneumococcus-like colonies are individually tested in qPCR for *piaB* and/or *lytA* and confirmed to be *S. pneumoniae* based on susceptibility to optochin (Miellet et al. 2022).

**Supplemental References**

ALMEIDA, S. T., PAULO, A. C., FROES, F., DE LENCASTRE, H. & SA-LEAO, R. 2021. Dynamics of Pneumococcal Carriage in Adults: A New Look at an Old Paradigm. *J Infect Dis,* 223**,** 1590-1600.

BLAND, J. M. & ALTMAN, D. G. 1986. Statistical methods for assessing agreement between two methods of clinical measurement. *Lancet,* 1**,** 307-10.

BOELSEN, L. K., DUNNE, E. M., GOULD, K. A., RATU, F. T., VIDAL, J. E., RUSSELL, F. M., MULHOLLAND, E. K., HINDS, J. & SATZKE, C. 2020. The Challenges of Using Oropharyngeal Samples To Measure Pneumococcal Carriage in Adults. *mSphere,* 5.

BUSTIN, S. A., BENES, V., GARSON, J. A., HELLEMANS, J., HUGGETT, J., KUBISTA, M., MUELLER, R., NOLAN, T., PFAFFL, M. W., SHIPLEY, G. L., VANDESOMPELE, J. & WITTWER, C. T. 2009. The MIQE guidelines: minimum information for publication of quantitative real-time PCR experiments. *Clin Chem,* 55**,** 611-22.

GIAVARINA, D. 2015. Understanding Bland Altman analysis. *Biochem Med (Zagreb),* 25**,** 141-51.

KOO, T. K. & LI, M. Y. 2016. A Guideline of Selecting and Reporting Intraclass Correlation Coefficients for Reliability Research. *J Chiropr Med,* 15**,** 155-63.

KRONE, C. L., WYLLIE, A. L., VAN BEEK, J., ROTS, N. Y., OJA, A. E., CHU, M. L., BRUIN, J. P., BOGAERT, D., SANDERS, E. A. & TRZCINSKI, K. 2015. Carriage of Streptococcus pneumoniae in aged adults with influenza-like-illness. *PLoS One,* 10**,** e0119875.

LENNON, J. T., MUSCARELLA, M. E., PLACELLA, S. A. & LEHMKUHL, B. K. 2018. How, When, and Where Relic DNA Affects Microbial Diversity. *mBio,* 9.

MCGRAW, K. O. & WONG, S. P. 1996. Forming inferences about some intraclass correlation coefficients. *Psychological methods,* 1**,** 30.

MCHUGH, M. L. 2012. Interrater reliability: the kappa statistic. *Biochem Med (Zagreb),* 22**,** 276-82.

MIELLET, W. R., MARIMAN, R., PLUISTER, G., DE JONG, L. J., GRIFT, I., WIJKSTRA, S., VAN LOGCHEM, E. M., VAN VELDHUIZEN, J., IMMINK, M. M., WIJMENGA-MONSUUR, A. J., ROTS, N. Y., SANDERS, E. A. M., BOSCH, T. & TRZCINSKI, K. 2021. Detection of Neisseria meningitidis in saliva and oropharyngeal samples from college students. *Sci Rep,* 11**,** 23138.

MIELLET, W. R., VAN VELDHUIZEN, J., LITT, D., MARIMAN, R., WIJMENGA-MONSUUR, A. J., BADOUX, P., NIEUWENHUIJSEN, T., THOMBRE, R., MAYET, S., ELETU, S., SHEPPARD, C., VAN HOUTEN, M. A., ROTS, N. Y., MILLER, E., FRY, N. K., SANDERS, E. A. M. & TRZCINSKI, K. 2022. It Takes Two to Tango: Combining Conventional Culture With Molecular Diagnostics Enhances Accuracy of Streptococcus pneumoniae Detection and Pneumococcal Serogroup/Serotype Determination in Carriage. *Front Microbiol,* 13**,** 859736.

MIELLET, W. R., VAN VELDHUIZEN, J., LITT, D., MARIMAN, R., WIJMENGA-MONSUUR, A. J., NIEUWENHUIJSEN, T., CHRISTOPHER, J., THOMBRE, R., ELETU, S., BOSCH, T., ROTS, N. Y., VAN HOUTEN, M. A., MILLER, E., FRY, N. K., SANDERS, E. A. & TRZCIŃSKI, K. 2023. A Spitting Image: Molecular Diagnostics Applied to Saliva Enhance Detection of Streptococcus pneumoniae and Pneumococcal Serotype Carriage. *(submitted)*.

MIELLET, W. R., VAN VELDHUIZEN, J., NICOLAIE, M. A., MARIMAN, R., BOOTSMA, H. J., BOSCH, T., ROTS, N. Y., SANDERS, E. A. M., VAN BEEK, J. & TRZCINSKI, K. 2020. Influenza-like Illness Exacerbates Pneumococcal Carriage in Older Adults. *Clin Infect Dis*.

RAMAKERS, C., RUIJTER, J. M., DEPREZ, R. H. & MOORMAN, A. F. 2003. Assumption-free analysis of quantitative real-time polymerase chain reaction (PCR) data. *Neurosci Lett,* 339**,** 62-6.

RANGANATHAN, P., PRAMESH, C. S. & AGGARWAL, R. 2017. Common pitfalls in statistical analysis: Measures of agreement. *Perspect Clin Res,* 8**,** 187-191.

ROGERS-BROADWAY, K. R. & KARTERIS, E. 2015. Amplification efficiency and thermal stability of qPCR instrumentation: Current landscape and future perspectives. *Exp Ther Med,* 10**,** 1261-1264.

RUIZ-VILLALBA, A., RUIJTER, J. M. & VAN DEN HOFF, M. J. B. 2021. Use and Misuse of Cq in qPCR Data Analysis and Reporting. *Life (Basel),* 11.

SATZKE, C., TURNER, P., VIROLAINEN-JULKUNEN, A., ADRIAN, P. V., ANTONIO, M., HARE, K. M., HENAO-RESTREPO, A. M., LEACH, A. J., KLUGMAN, K. P., PORTER, B. D., SA-LEAO, R., SCOTT, J. A., NOHYNEK, H., O'BRIEN, K. L. & GROUP, W. H. O. P. C. W. 2013. Standard method for detecting upper respiratory carriage of Streptococcus pneumoniae: updated recommendations from the World Health Organization Pneumococcal Carriage Working Group. *Vaccine,* 32**,** 165-79.

SHROUT, P. E. & FLEISS, J. L. 1979. Intraclass correlations: uses in assessing rater reliability. *Psychological bulletin,* 86**,** 420.

TELLINGHUISEN, J. & SPIESS, A. N. 2019. qPCR data analysis: Better results through iconoclasm. *Biomol Detect Quantif,* 17**,** 100084.

TRZCINSKI, K., BOGAERT, D., WYLLIE, A., CHU, M. L., VAN DER ENDE, A., BRUIN, J. P., VAN DEN DOBBELSTEEN, G., VEENHOVEN, R. H. & SANDERS, E. A. 2013. Superiority of trans-oral over trans-nasal sampling in detecting Streptococcus pneumoniae colonization in adults. *PLoS One,* 8**,** e60520.

WYLLIE, A. L., CHU, M. L., SCHELLENS, M. H., VAN ENGELSDORP GASTELAARS, J., JANSEN, M. D., VAN DER ENDE, A., BOGAERT, D., SANDERS, E. A. & TRZCINSKI, K. 2014. Streptococcus pneumoniae in saliva of Dutch primary school children. *PLoS One,* 9**,** e102045.

WYLLIE, A. L., WIJMENGA-MONSUUR, A. J., VAN HOUTEN, M. A., BOSCH, A., GROOT, J. A., VAN ENGELSDORP GASTELAARS, J., BRUIN, J. P., BOGAERT, D., ROTS, N. Y., SANDERS, E. A. M. & TRZCINSKI, K. 2016. Molecular surveillance of nasopharyngeal carriage of Streptococcus pneumoniae in children vaccinated with conjugated polysaccharide pneumococcal vaccines. *Sci Rep,* 6**,** 23809.

**
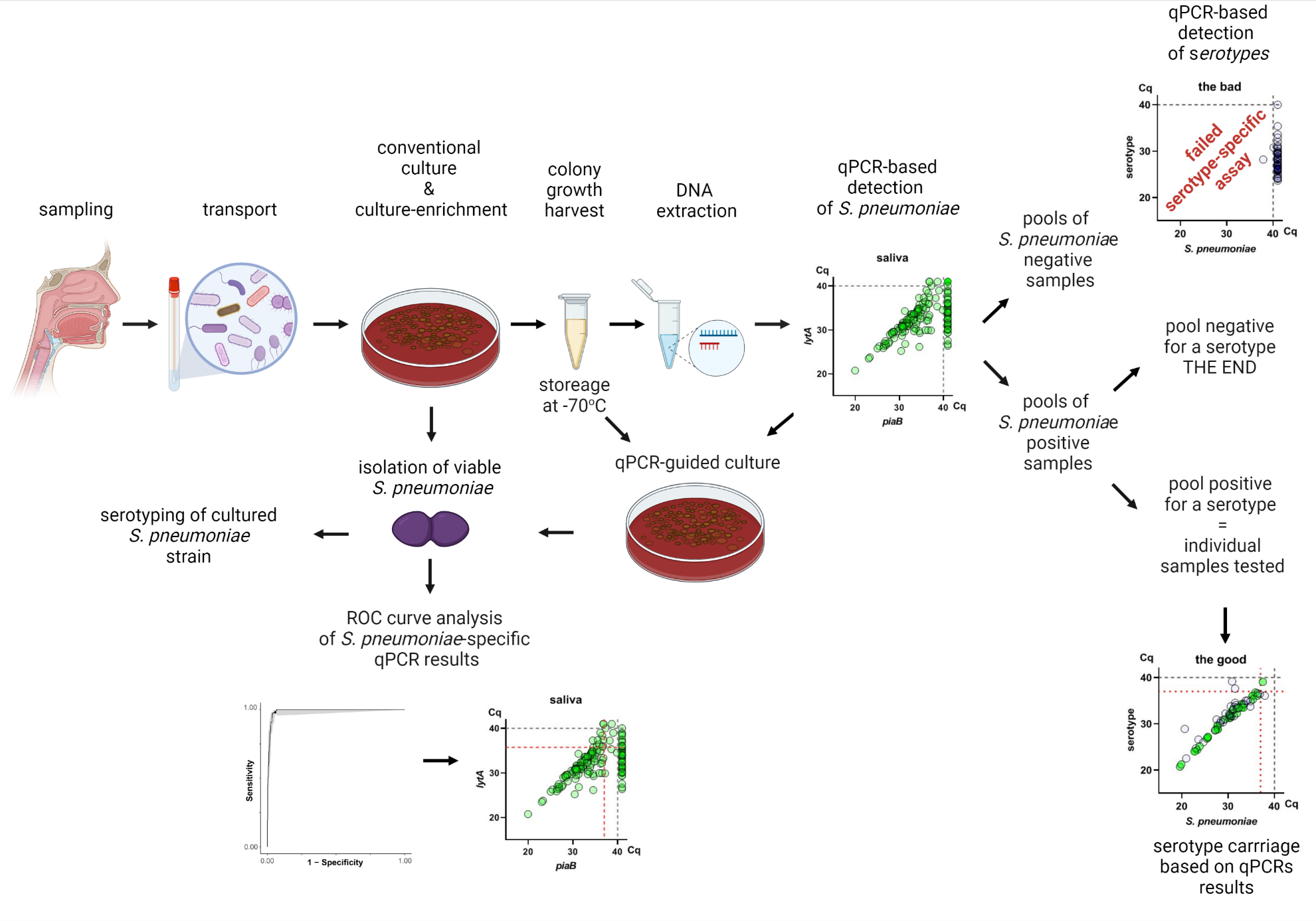
**

**FIGURE S1.** **Visual summary of the “optimal” protocol.** A nasopharyngeal, oropharyngeal or saliva sample is collected from a study participant and transported in medium supplemented with 10% glycerol. The sample is cultured on defibrinated sheep blood agar supplemented with gentamicin. Presumptive *S. pneumoniae* colonies are isolated for detection by conventional culture. All microbial growth on the culture plate is harvested and stored in medium supplemented with 10% glycerol. DNA extraction is performed on the culture-enriched harvest and molecular detection is conducted using a dual-target approach with *lytA* and *piaB* or *lytA* and SP2020 (*bguR*). Concordance between quantified qPCR targets is evaluated and samples are classified as positive or negative by qPCR. Receiver operating characteristic curve analysis is conducted with culture as reference in order to calculate data-driven C_q_ thresholds. Nasopharyngeal and oropharyngeal samples positive by qPCR for *S. pneumoniae* yet negative by conventional culture are subjected to qPCR-guided culturing. Samples negative for *S. pneumoniae* by qPCR are pooled by ten and positive samples are pooled by five. Pools are tested with serotype/serogroup-specific qPCR assays. The specificity of serotype/serogroup-specific assays is evaluated using pools from negative samples. Pools from positive samples are tested for serotype/serogroup-specific qPCR assays, when a pool is positive for a particular assay all samples from that pool are individually tested. Concordance between serotype/serogroup-specific qPCR quantification and *piaB* or *lytA* is evaluated. The figure was made in BioRender.


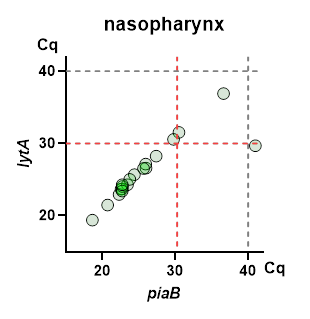

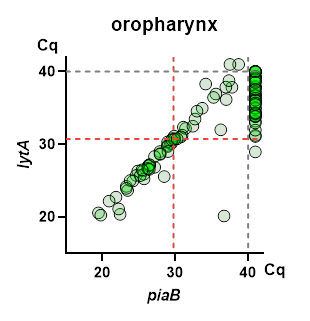

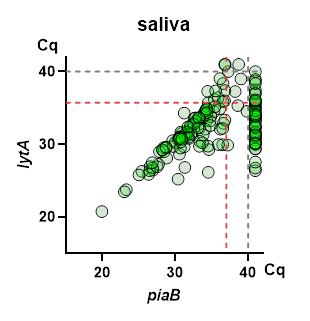


**FIGURE S2.** **Detection of *Streptococcus pneumoniae* with molecular methods in nasopharyngeal, oropharyngeal and saliva samples collected from n=322 community dwelling asymptomatic adults in The Netherlands in 2014/2015** (Miellet et al., 2023)**.** Panels depict scatter plots of *piaB* and *lytA* qPCR cycle threshold (C_q_) values for three different types of samples. Each symbol represents an individual sample. Samples with a C_q_ >40 for both *pia*B and *lytA* have been excluded.

**
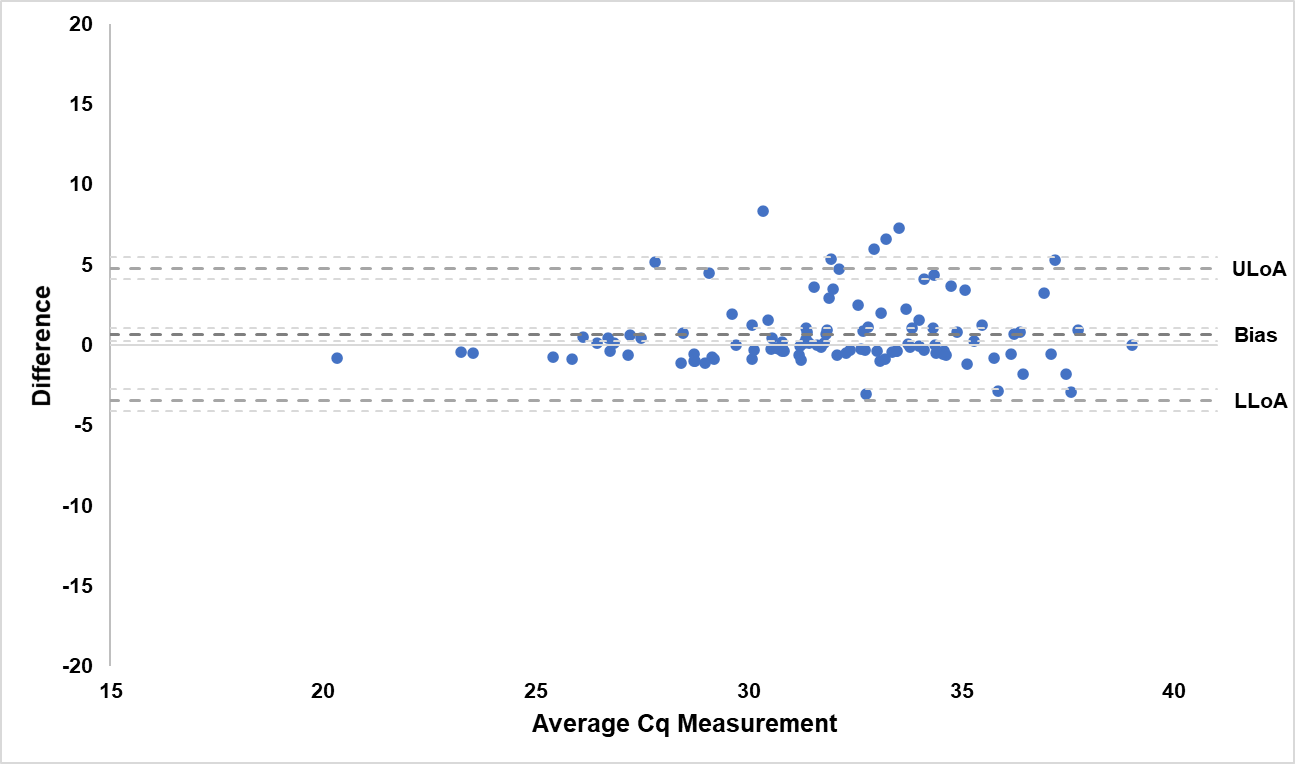
**

**FIGURE S3.** **Bland-Altman plot for *piaB* and *lytA* data from *S. pneumoniae* positive saliva samples of adults** (Miellet et al., 2023)**.** On the the y-axis the difference in C_q_ between *piaB* and *lytA* is depicted and on the x-axis the average C_q_ value from samples are shown. The mean difference (Bias) between *piaB* and *lytA* _Cq_ measurements is depicted (bias) and the upper and lower 95% confidence intervals are shown as upper limit of agreement (ULoA) and lower limit of agreement (LLoA), respectively.
